# Supplementary material for: Mannitol Is a Good Anticaking Agent for Spray-Dried Hydroxypropyl-Beta-Cyclodextrin Microcapsules
Source: Molecules. 2023 Jan 22;28(3):1119. doi: 10.3390/molecules28031119 (PMC9921659; doi:10.3390/molecules28031119)
Supplement: Supplementary file 1 [file molecules-28-01119-s001.zip › molecules-2083663-supplementary.pdf]

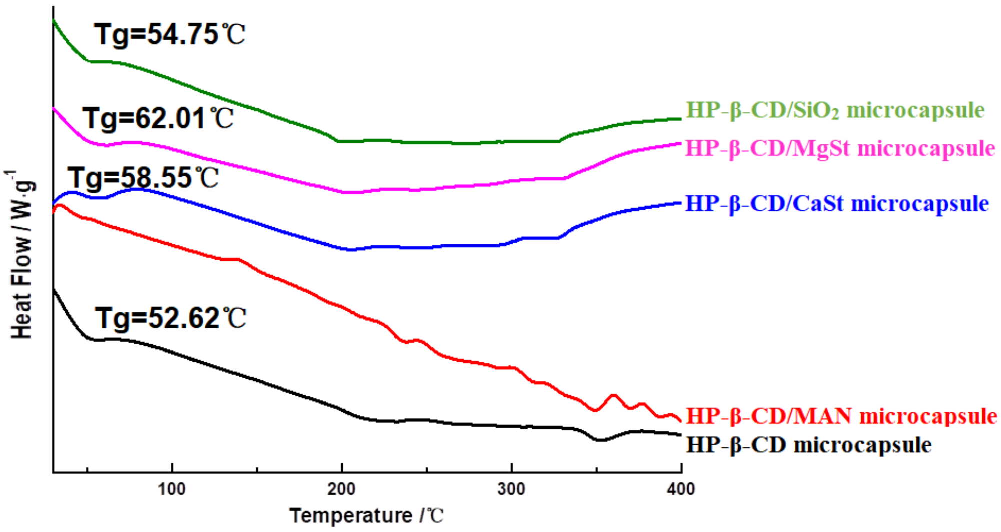

Figure S1: Differential scanning calorimeter patterns of microcapsules with anticaking agents
